# Angiotensin Converting Enzyme 2 (ACE2) Expression in the Aged Brain and Visual System

**Published:** 2021-09-30

**Authors:** James M. Hill, Christian Clement, L. Arceneaux, Walter J. Lukiw

**Affiliations:** 1 LSU Neuroscience Center, Louisiana State University Health Sciences, New Orleans, USA; 2 Department of Ophthalmology, Louisiana State University Health Sciences Center, New Orleans, USA; 3 Department of Pharmacology, LSU Health Science Center, New Orleans, USA; 4 Department of Microbiology, LSU Health Science Center, New Orleans, USA; 5 Experimental Therapeutics and Human Toxicology Lab, Southern University, New Orleans, USA; 6 Department of Neurology, Louisiana State University Health Sciences Center, New Orleans, USA

**Keywords:** Alzheimer’s disease, angiotensin-converting enzyme 2 (ACE2), Occipital lobe, Ocular cells, Optic nerve, retinal pigment epithelial (RPE) cells, severe acute respiratory syndrome coronavirus 2 (SARS-CoV-2)

## Abstract

Multiple lines of evidence currently indicate that the severe acute respiratory syndrome coronavirus 2 (SARS-CoV-2) gains entry into human host cells *via* a high-affinity interaction with the angiotensin-converting enzyme 2 (ACE2) transmembrane receptor. Research has further shown the widespread expression of the ACE2 receptor on the surface of many different immune, non-immune and neural host cell types, and that SARS-CoV-2 has the remarkable capability to attack many different types of human-host cells simultaneously. One principal neuroanatomical region for high ACE2 expression patterns occurs in the brainstem, an area of the brain containing regulatory centers for respiration, and this may in part explain the predisposition of many COVID-19 patients to respiratory distress. Early studies also indicated extensive ACE2 expression in the whole eye and the brain’s visual circuitry in aged humans. In this study we analyzed ACE2 receptor expression at the mRNA and protein level in multiple cell types involved in human vision, including cell types of the external eye and several deep brain regions known to be involved in the processing of visual signals. Here we provide evidence: (**i**) that many different optical and neural cell types of the human visual system provide receptors essential for SARS-CoV-2 invasion; (**ii**) of the remarkable ubiquity of ACE2 presence in cells of the eye and anatomical regions of the brain involved in visual signal processing; (**iii**) that ACE2 receptor expression in different ocular cell types and visual processing centers of the brain provide multiple compartments for SARS-CoV-2 infiltration; and (**iv)** of a gradient of increasing ACE2 expression from the anterior surface of the eye to the visual signal processing areas of the occipital lobe and the primary visual neocortex. A gradient of ACE2 expression from the eye surface to the occipital lobe may provide the SARS-CoV-2 virus a novel pathway from the outer eye into deeper anatomical regions of the brain involved in vision. These findings may explain, in part, the many recently reported neuro-ophthalmic manifestations of SARS-CoV-2 infection in COVID-19 affected patients.

## INTRODUCTION

The coronavirus disease 2019 (COVID-19) pandemic originated late in the year 2019 in an open air market in the city of Wuhan in China’s Hubei province. Early reports suggested that the emergence of SARS-CoV-2 may have occurred via ‘zoonotic spillover’ from the fruit bat (*Pteropus scapulatus*) and/or other mammalian and/or avian vectors and an interspecies viral transmission [1–6]. Three key findings on the mechanism of SARS-CoV-2 infection were that: (i) direct contact of the novel enveloped, positive single-stranded RNA (ssRNA) SARS-CoV-2 beta coronavirus that causes COVID-19 with host cell surface receptors is the primary suspected route of transmission; (ii) the SARS-CoV-2 virus, via its surface spike (S1) protein requires the single-pass angiotensin-converting enzyme 2 (ACE2) transmembrane receptor to gain entry into human host cells; and (iii) that ACE2, a zinc containing surface receptor protein and metallo-carboxypeptidase (EC 3.4.17.23) normally involved as a key component of the renin-angiotension system (RAS), is ubiquitously expressed in many different human cell types. Immuno-histochemical analysis has recently confirmed expression of ACE2 at the protein level in alveolar and epithelial cells of the respiratory system, enterocytes and intestinal epithelial and endothelial cells, kidney cells of the renal tubule and immune cells, such as alveolar monocytes and macrophages, and many different cell types of the CNS including those of the cerebral cortex, brainstem, the conjunctival epithelium of the eye, the optic nerve, cornea, the corneal epithelial surface and multiple cell types of the brain involved in vision and visual signal processing [5–12]. This may in part explain SARS-CoV-2-mediated invasion of a very wide spectrum of different host cell types including sensory and support cells of the eye and specific anatomical regions of the brain. This viral-mediated elicitation of a multipronged attack underscores the ubiquity, severity and extensive variety of signs and symptoms observed in COVID-19 patients.

Most recently emerging observations indicate widespread neuro-ophthalmic disruptions in COVID-19 infected patients [13–26]. Coronaviruses are known for their neurotropism towards brain cells and cells of the visual system, and COVID-19 infection is currently positively associated with ocular abnormalities including blurred vision, kerato-conjunctivitis (an inflammation of the conjunctiva), conjunctival hyperemia, chemosis (a swelling of the conjunctiva) [13–16], epiphora (increased tear secretions), anosmia, diplopia (monocular or binocular), acute-onset vision loss, acquired cortical blindness caused by damage to the brain’s occipital cortex in both primary and secondary brain visual processing centers [15–18], progressive monocular and binocular/bilateral blindness, eye pain with photophobia, eye pain with disturbances in extraocular movements and cranial nerve involvement [17–20], decreased visual acuity, optic neuritis, visual-associated disturbances in balance and gait issues, total or partial loss of vision in an otherwise normal-appearing eye [19–22], acute uveitis and other neurologic-ophthalmic symptoms including cranial neuropathies in the visual circuitry and a virally-induced Miller-Fisher syndrome [13–15,19–25]. COVID-19 infection is further associated: (i) with alterations in the ganglion cell and plexiform cell layers of the retina and reduced vessel density of the retinal capillary plexus as evidenced by using non-invasive imaging techniques including optical coherence tomography (OCT) angiography [15,26]; and (ii) the observation of increased secretion of tear fluids from the eye (epiphora) during COVID infection; ocular biofluids are known to contain viral particles and have been implicated in the passive transmission of COVID-19 via the nasolacrimal duct through the nasal cavity and into susceptible ACE2 receptor-enriched cells of the upper respiratory tract (see below) [6,20,23,24].

Because of the extraordinary and singular importance of the ACE2 transmembrane receptor in the recognition, attachment and entry of SARS-CoV-2 into host cells and requisite for SARS-CoV-2 infection, in the current report we investigated the expression of ACE2 at the messenger RNA (mRNA) and protein level in 20 different types of cells involved in human vision, including 11 cell types of the anterior eye and 9 brain regions involved in the processing of visual signals employing a novel highly sensitive radiolabel-hybridization-based detection system using gamma ^32^P-adenosine tri-phosphate ([γ-^32^P]dATP) radiolabeled ACE2 probes; see below [6,27,28]. In our 52 years of experience of researching gene expression abundance, speciation and complexity in the human brain and eye in our hands this technique has been found to be the most accurate and quantitative abundance-based analysis [6,27,28]. ACE2 protein levels were also assayed in 14 different eye cell types and anatomical regions of the brain involved in visual signal processing. As discussed more fully below, major findings include: (i) a very strong correlation between ACE2 mRNA abundance with ACE2 protein abundance in the eye and brain cells studied; (ii) the observation of detectable levels of ACE2 receptor expression found throughout eye cells and anatomical regions of the human brain involved in visual signal processing; (iii) that many different cell and tissue types of the visual system and brain provide multiple points of potential SARS-CoV-2 viral entry and host cell invasion and infectivity; and (iv) that a downhill gradient of ACE2 receptor abundance on ocular and brain cell membrane surfaces may, in part, favor translocation of the SARS-CoV-2 coronavirus into deeper anatomical regions of the visual pathways. The ubiquity, abundance and positioning of the ACE2 receptor throughout the visual system of the eye and brain may have implications relevant to the increasing number of reports linking SARS-CoV-2-association with visual disturbances and dysfunction and neuro-ophthalmic manifestations in COVID-19 patients.

## MATERIALS AND METHODS

### ACE2 mRNA analysis

**Figure 1:**
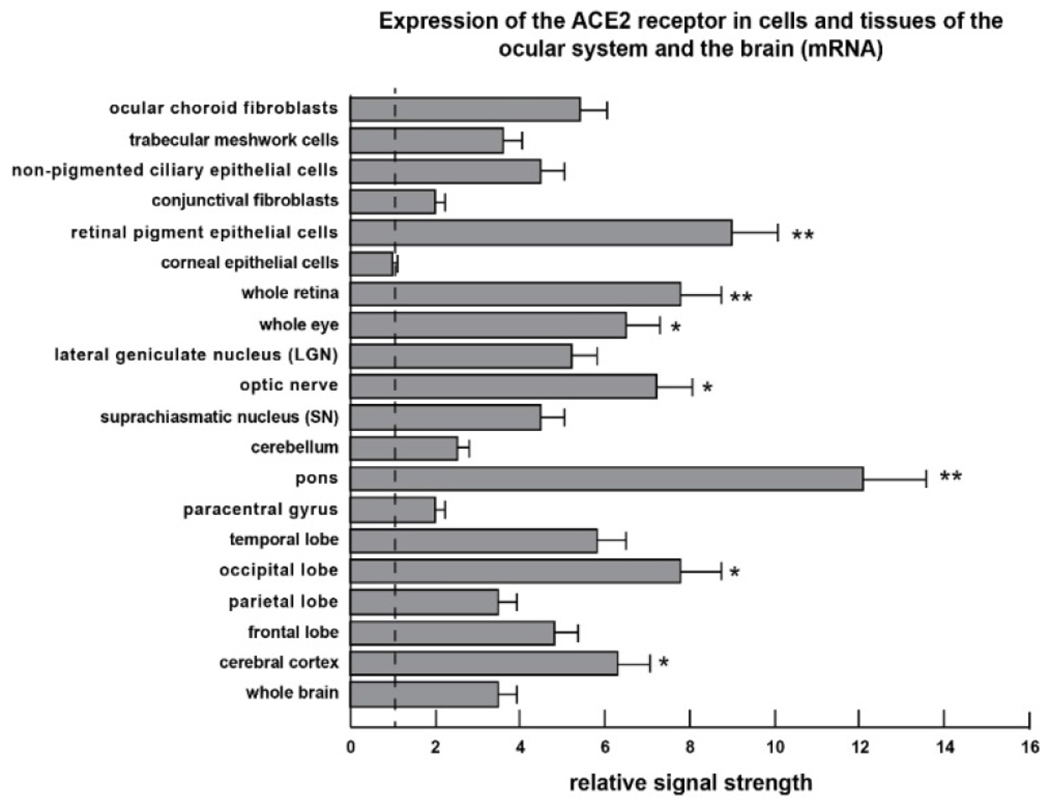
Cell- and tissue-specific patterns of ACE2 expression (mRNA) in human ocular cells and in selected areas of the brain involved in visual processing. The extent of ACE2 transmembrane receptor expression in the plasma membrane is an important indicator of ACE2 gene function, the susceptibility to SARS-CoV-2 invasion and the development of COVID-19. Significant ACE2 receptor expression was initially observed in the whole brain, whole eye and whole retina and subsequently cells and tissues involved in visual signal acquisition and processing were analyzed for ACE2 abundance. ACE2 acts as a receptor for the spike (S1) glycoprotein of the human coronavirus HCoV-NL63 and the human SARS-CoV and SARS-CoV-2 virus that causes COVID-19 (see: https://www.ncbi.nlm.nih.gov/gene?Db=gene&Cmd=DetailsSearch&Term=59272; last accessed 9 September 2021); in the mRNA experiments both G3PDH and β-actin DNA probes were used as abundance controls; in this experiment relative signal strength refers to control β-actin mRNA levels in the same tissues [6]; in this and one previous study [6] the highest expression of the SARS-CoV-2 ACE-2 receptor was found in the cerebral cortex and the occipital lobe, the pons and medulla oblongata of the brain and in the whole retina, the optic nerve and the ocular choroid and RPE cells of the eye. High expression of the ACE2 receptor in the eye and neural pathways involved in vision and visual processing may predispose this circuitry of the visual system to attracting SARSCoV-2 and viral invasion. A vertical dashed line at 1.0 is included for ease of comparison; a minimum of N=3 HyBond N+ or MTE filters were used for each tissue determination; *p<0.05; **p<0.01 (ANOVA); error bars represent one standard deviation of the mean.

The entire analytical protocol has been recently reported and updated in some detail by our laboratories [6,27,28]. Briefly, human multiple tissue expression (MTE) array panels (Invitrogen-Clontech, Palo Alto CA; cat no. 7775-1) or eye and brain cells or tissues were utilized to analyze brain- or visual-relevant expression of ACE2 receptor mRNA. The MTE arrays utilized contain polyA+ mRNA samples (each dot-shaped sector containing ^~^2.0 μg of polyA+ RNA) enriched from 9 human brain regions with 8 independent DNA and RNA controls where the ACE2 receptor is not expressed; polyA+ RNA samples were further extracted from ocular cells (including epithelial, conjunctival, keratocytes (corneal fibroblasts), iris fibroblasts, retinal pigment epithelial (RPE) cells and trabecular meshwork cells obtained from ScienCell (https://www.sciencellonline.com/productsservices/primary-cells/human/cell-systems/ocular-cell-system.html) or were kind gifts from Dr. PN Alexandrov (Figure 1).

Eye and brain tissue-sourced mRNA were obtained from pools of aged control eye and brain tissue sources [6,27,28]. All postmortem tissues utilized in this study had a mean age and one standard deviation of 68.5 +/− 12.2 yrs. RNA concentrations were quantified using a Agilent 2100 Bioanalyzer and Agilent test chips (ThermoFisher Scientific, Waltham MA, USA) and ^~^2.0 to ^~^5.0 μg polyA+ RNA were spotted onto HyBond N+ nylon membrane filters using the manufacturer’s protocols as previously described (ThermoFisher Scientific) [6,27,28]; Alexandrov et al., 2012; Tukiw et al., 2020). A unique human-specific 26 nucleotide (nt) ACE2 receptor-specific DNA probe 5′-CTTGCAGCTACACCAGTTCCCAGGCA-3′ (US NIH/NLM Sequence ID: AY217547.1; https://blast.ncbi.nlm.nih.gov/Blast.cgi#27978647; see also accession AB046569.1; https://www.ncbi.nlm.nih.gov/nuccore/AB046569.1); last accessed 9 September 2021) exhibited no homology to ACE1 [6,28–30]; while other ACE2-specific probes have been used previously that gave almost identical hybridization signals, data for the 26 nt ACE2 DNA probe is presented in this report. The 26 nt ACE2 receptor DNA probe was designed so that the sequence crosses two exons, thereby detecting only mature ACE2 mRNA and not ACE2 genomic DNA or heterogeneous RNA (hnRNA) containing the ACE2 DNA or RNA sequence. DNA probes were used due to their stability (as compared to unprotected RNA probes) and RNA-DNA hybrids are known to elicit a more accurate and stable hybridization with a higher energy of association (E_A_) between the ACE2 DNA probe and the membrane-bound polyA+ mRNA target [6,31,32]; https://www.ncbi.nlm.nih.gov/gene?Db=gene&Cmd=DetailsSearch&Term=59272; last accessed 9 September 2021). In these experiments ACE2 DNA probes were end-labeled by using [γ-^32^P]dATP (3,000 Ci/mmol, Amersham Redivue™); either glyceraldehyde 3-phosphate dehydrogenase (G3PDH) or beta-actin (β-actin) were used for control mRNA normalization normalization (see http://www.takara.co.kr/file/manual/pdf/PT3307-1.pdf [6,26–29,32]. To obtain maximal signal quantitation accuracy hybridized and washed membranes were overlaid on the previously marked MTE or polyA+ RNA spotted HyBond N+ nylon templates, excised and each sector counted separately in 10 ml liquid scintillation fluid (Ultima Gold, PerkinElmer, Waltham MA) using a LS600016 LSC Liquid Scintillation System, Beckman, Fullerton CA); relative signal intensities were expressed by comparing the ACE2 hybridization signal to the G3PDH and/or β-actin signal for each polyA+ mRNA on the MTE or polyA+ array [6,28–35].

### ACE2 protein analysis

**Figure 2:**
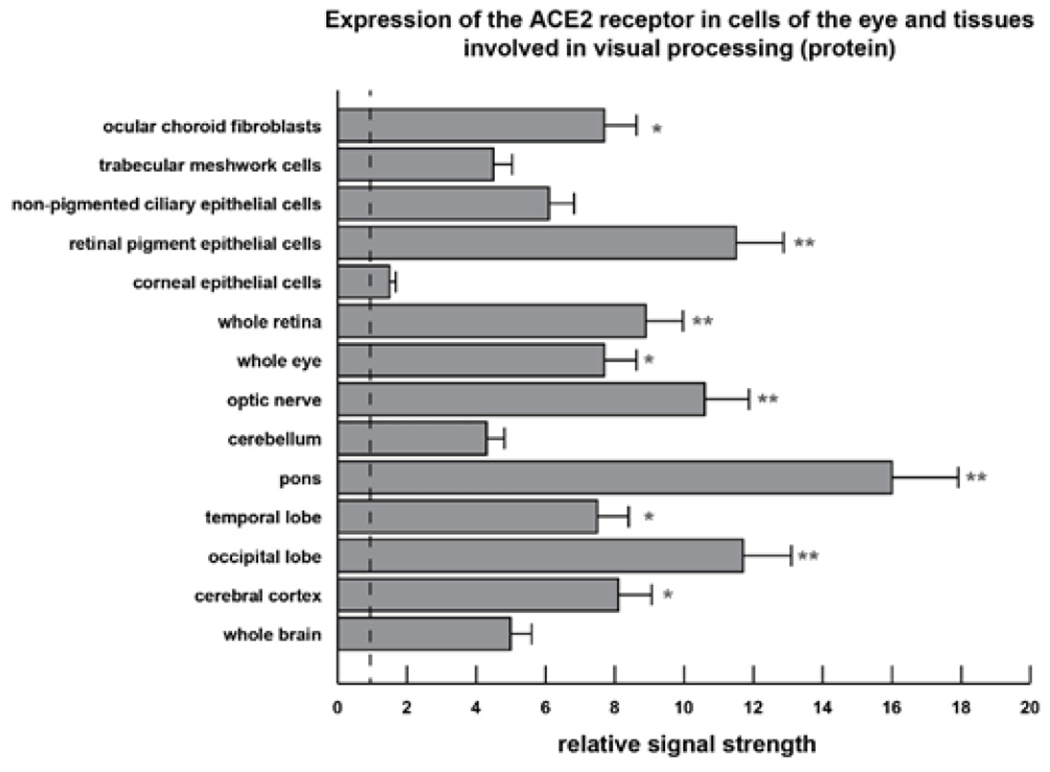
Cell- and tissue-specific patterns of ACE2 expression at the protein level in selected human ocular cells and in anatomical areas of the brain involved in visual processing; bar graph of ELISA analysis (see Supplementary File 1); we observed a very strong correlation between ACE2 mRNA abundance (Figure 1) with ACE2 protein abundance (Figure 2); the highest expression of the SARS-CoV-2 receptor ACE-2 protein was found in RPE cells, the whole retina, optic nerve, the pons and the occipital lobe that contains the primary visual cortex and main visual processing area (Brodmann Area17); relative signal strength refers to control β-actin protein levels in the same tissues; see text for further details; a vertical dashed line at 1.0 is included for ease of comparison; a minimum of N=3 ELISA analyses were performed for each protein determination in cells or tissues; *p<0.05; **p<0.01 (ANOVA); error bars represent one standard deviation of the mean.

Protein extracts from cells and/or tissues involved in human vision, including ocular choroidal fibroblasts, trabecular meshwork cells, non-pigmented ciliary epithelial cells, retinal pigment epithelial cells, corneal epithelial cells, whole retina, whole eye and tissues from brain visual processing pathways including the optic nerve, cerebellum, pons, temporal lobe, occipital lobe (Brodmann Area 17, containing the primary visual cortex), cerebral cortex and whole brain were generated using a ProteoExtract Complete Mammalian Proteome Extraction Kit (cat no. 539779, Calbiochem/Millipore-Sigma Burlington MA) and were assayed for protein concentration using a Non-Interfering Protein Assay kit (cat no. 488250, Calbiochem/Millipore-Sigma) at 480 nm; protein samples were stored in at −81°C according to the manufacturers protocol (Millipore-Sigma); ACE2 protein [UniProtKB - Q9BYF1 (ACE2_HUMAN)] abundance these selective cell extracts of the visual system and brain were analyzed using a quantitative colorimetric (450 nm) sandwich ELISA specific for human ACE2 using a Fluoroskan Ascent FT Microplate Fluorometer and Luminometer (Cat no. 5200220, ThermoFisher Scientific, Waltham MA; sensitivity 1052 pg/ml; detection range 1.5 ng/ml - 255 ng/ml (Human ACE2 ELISA Kit ab235649; Abcam Cambridge MA, USA); human recombinant ACE2 protein (Abcam ab151852) and human beta-actin (β-actin; anti-beta actin antibody (Abcam ab8227) were used as internal controls to quantify relative ACE2 and β-actin protein abundance in each sample according to the manufacturer’s instructions (Figure 2); concentrations of ACE2 were measured in triplicate and interpolated from the ACE2 standard curve and corrected for sample dilution as according to manufacturer’s protocol; human ACE2 was expressed as ng/ml (Abcam; https://www.abcam.com/human-ace2-elisa-kit-ab235649.html; last accessed 9 September 2021; see Supplementary file 1).

### Statistical significance

The analysis of statistical significance was evaluated using a two-way factorial analysis of variance (p, ANOVA; SAS Institute, Cary NC, USA). A p<0.05 (ANOVA) was deemed as statistically significant; a p<0.01 (ANOVA) was deemed as very highly significant; experimental values were expressed as the means ± one standard deviation (SD) of that mean (Figures 1 and 2).

## RESULTS

In the current study all ocular and brain cell types and tissues exhibited easily detectable ACE2 receptor signals, and in agreement with previous reports underscore the ubiquitous nature of ACE2 receptor expression throughout the human visual system and CNS (Figures 1 and 2) [5,6,24]. Importantly, and as indicated in previous studies, negative controls, yeast and microbial RNA controls, synthetic homo-ribonucleotide polymers and random oligonucleotides showed no expression of the ACE2 receptor mRNA [6]. The highest expression of ACE2 mRNA in the brain was found in the brainstem region known as the pons, situated anterior to the cerebellum between the midbrain and medulla oblongata, and an important relay center known to conduct signals from the cerebrum, through to the cerebellum and medulla oblongata, including fiber tracts that involve the transmission of visual sensory signals into the thalamus [36,37]. The pons is known to contain neural circuits that deal primarily with the regulation of respiration, taste (aguesia), audition, saccadic eye movement, facial sensation and expression and equilibrium, all of which are known to be affected or disrupted in COVID-19 disease [7,17,24,25,36–38]. The respiratory tract connects to the brain without the protection of a blood–brain barrier, and that SARS-CoV-2 might in the early invasive phase attack the cardiorespiratory regulatory nodes located in the pons and medulla oblongata, giving rise to both respiratory and cardiac disruption, as is also commonly observed in COVID-19 patients [25,38–39]. The highest expression of the ACE2 receptor protein in the ocular cells and brain tissues involved in visual signaling and examined in this study were found in ocular choroid fibroblasts and retinal pigment epithelial (RPE) cells, a monolayer of pigmented cells of neuro-ectodermal origin situated between the neurosensory retina and the choroid that nourishes the visual photoreceptor cells.

We further note the relatively high expression of the ACE2 receptor mRNA and protein throughout the association neocortex and especially the occipital lobe of the brain that contains the primary visual processing areas. Interestingly ACE2 receptor expression at the protein level was in the order of the corneal epithelial cells < trabecular meshwork cells << non-pigmented ciliary epithelial cells < ocular choroid fibroblasts < whole retina << optic nerve << occipital lobe <<< pons, indicating an increasing ACE2 expression gradient along a pathway from the exterior of the eye (i.e. the anterior sensory aspect of the human visual system) that is exposed to the environment into to deeper vision-relevant regions of the association neocortex located in the occipital lobe (Brodmann area 17; the primary visual cortex).

## DISCUSSION

At 29,811 nucleotides the unusually large SARS-CoV-2 virus possesses an enveloped, positive-stranded ssRNA genome that shares significant homology to previously described SARS-CoV and the middle east respiratory syndrome coronavirus (MERS-CoV) [40–42]. SARS-CoV-2 has an extremely high binding affinity for a ubiquitous 92.5 kDa, zinc-containing, membrane-integral ACE2 cell surface receptor (EC 3.4.17.23). SARS-CoV-2-ACE2 binding results in the endocytosis and translocation of both SARS-CoV-2 and the ACE2 receptor into the endosomes of infected cells [5,7,9,22,43–47]. Other ACE2-associated proteins such as the serine protease 2 TMPRSS2 (epitheliasin; EC 3.4.21.109) may facilitate SARS-CoV-2 entry [11,43,48]. Importantly, ACE2 receptor gene expression, both at the level of mRNA and protein is detectable in all eye and brain cells and tissues so far examined and underscores the potential for SARS-CoV-2 entry and infectivity into a remarkably wide variety of host cells populating the human visual and central nervous systems.

Many neurotropic viruses that exhibit ocular tropism, such as the double-stranded DNA (dsDNA) virus herpes simplex 1 (HSV-1) and the ssRNA virus SARS-CoV-2, are also abundant in shed tears and ocular secretions from infected persons [23,24,49–55]. Vital for the health of the outer eye, tear production and drainage via the nasolacrimal duct may provide another novel conduit for SARS-CoV-2 translocation from the eye to the nasal cavity and into the upper respiratory tract that is enriched in ACE2 receptors [23,24,55]. As suggested by the current data another available route of SARS-CoV-2 entry and proliferation in the eye and brain may be via structural support and sensory cells of the eye, through the optic nerve across the optic chiasm to the lateral geniculate nucleus (LGN) and via optic radiations on into the primary visual cortex located in the occipital lobe (Brodmann area 17) of the brain (Figures 1 and 2).

Currently the pathophysiology and ocular pathomechanism for the transmission of SARS-CoV-2 remains an understudied area of viral and retinal neurobiology, and even basic questions, like the initial incapacitating effects of the potent glycoside hydrolase lysozyme (EC 3.2.1.17), a major component of the tear biofluid, on SARS-CoV-2 viability and infectivity are not well understood. Interestingly, ACE2 receptor abundance has been shown to be altered in progressive neurological disorders that include Alzheimer’s disease (AD), but whether or not AD patients have altered susceptibility to SARS-CoV-2 infectivity is another understudied research area [54,55]. Fortunately, the human eye has developed a wide array of defense mechanisms against microbial infection and has evolved a number of anti-microbial (both anti-bacterial and anti-viral) strategies to protect and preserve ocular surfaces and visual system structure and function. Firstly, the surface of the eye is directly exposed to the external environment and hence is susceptible to airborne microbial contamination from potentially pathogenic microbes including viruses, bacteria, fungi and parasites and their pathogen-associated molecules. Airborne contamination includes SARS-CoV-2, often as an aerosolized spray of microdroplets, and is the major known form of a highly infective SARS-CoV-2 viral transmission [24,51,53,56]. Besides different protective anti-microbial substances in the tear fluid such as mucins and globular glycoproteins such as lactoferrin, the tear mucosal film which coats the cornea and conjunctiva contains multiple anti-microbial components including lysozyme (also known as muramidase or N-acetylmuramide glycan-hydrolase), cationic anti-microbial peptides, surfactant protein-D, several RNAse enzymes, S100A peptides such as psoriasin (S100A7) and others, and these are important components of the innate-immune defense system of the eye providing protection against a wide range of potential airborne ocular pathogens [57,58; unpublished observations]. Interestingly, there is recent evidence that the ocular surface microbiota, the resident non-pathogenic symbiotic microorganisms that colonize the conjunctiva and cornea, and the microbiota of other areas of the body, such as the gastrointestinal (GI) tract and oral microbiome are involved in the development and pathophysiology of several ophthalmic diseases including the susceptibility to microbial, and in particular, viral transmission and infection [49–53,59]. Of further emerging interest are the effects of SARS-CoV-2 invasion on the biochemistry, molecular genetics and innate-immunity of neural and ocular host cells and the viral-mediated induction of post-transcriptional and pro-inflammatory signaling factors including certain microRNAs (miRNAs) that modulate the expression of genes involved in viral replication, neuro-inflammation, innate-immune signaling and progressive and age-related aspects of inflammatory neurodegeneration [50–53,59].

## SUMMARY

Our scientific understanding of the factors involved in SARS-CoV-2 viral transmission and affinity for multiple human host cell targets continues to evolve. There is now an expanding list of documented impairments of the human visual system and multiple types of visual dysfunction and/or vision deficiency associated with COVID-19 infection [15–25,46,51–53,60–64]. The current study provides new insight into the distribution of the ACE2 receptor in eye and brain cells and tissues involved in visual signal processing. The ACE2 receptor is acknowledged as the major molecular receptor for SARS-CoV-2, and specific anatomical regions of the eye and the visual circuitry of the brain contain abundantly detectable ACE2 receptor mRNA and protein. If the abundance of the ACE2 receptor has any bearing on the ability of attracting and binding SARS-CoV-2, allowing viral entry into human host cells, high ACE2 receptor expression along a gradient from the most exterior surface of the eye via multiple visual processing centers to the primary visual cortex in the occipital lobe provides another novel route for SARS-CoV-2 viral transmission and potential translocation into deeper anatomical regions of the visual brain [64].

In summary the remarkable ubiquity of ACE2 receptor expression in the trabecular meshwork and ocular choroid cells of the outer eye, RPE cells, the optic nerve and optic radiations to the occipital cortex suggests: (i) that multiple cell types of the visual system provide multiple potential entry points for SARS-CoV-2 invasion; (ii) that this broad spectrum of cell types involved in vision and potentially susceptible to SARS-CoV-2 infection reflects on the equally wide range of visual functions that may be impacted by this lethal virus; (iii) that a gradient of increasing abundance of ACE2 receptor expression in several different ocular cell types along the visual processing pathways of the brain provides SARS-CoV-2 access to anatomical regions of the brain involved in visual processing functions; and (iv) that as reflected by ACE2 abundance there are multiple potential compartments that support SARS-CoV-2 accessibility and infiltration in the human visual system. These results further indicate that under certain circumstances that eyeglasses or face-shields may be as important as face-masks in reducing the transmission and spread of the SARS-CoV-2 virus, and that COVID-19 healthcare workers and ophthalmologists may need to take additional care when dealing with both SARS-CoV-2 carriers and COVID-19 patients.

## Supplementary Material

1

## Data Availability

All data regarding ‘Methodology’ is available in the ‘Materials and Methods’ Section of the current manuscript. All relevant experimental data in this study are presented in 2 Figures and 2 Supplementary data files appended to this research communication. Any relevant queries on data, materials and methodology should be requested in writing and consultation with the corresponding author. We thank you for your interest in advance and would be happy to provide any ancillary information that you may need.
