# Supplementary material for: Angiotensin Converting Enzyme 2 (ACE2) Expression in the Aged Brain and Visual System
Source: J Aging Sci. Author manuscript; Available in PMC 2021 Nov 10. (PMC8580311)
Supplement: 1 [file NIHMS1750170-supplement-1.pdf]

SUPPLEMENTARY FILES

Supplementary File 1A: Standard curve of human ACE2 as analyzed using a human ACE2 ELISA Kit (ab235649; Abcam, Cambridge MA, USA; ELISA assay sensitivity is ~1052 pg/ml; range: 1.5 ng/ml - 255 ng/ml); based on the standard curve, mean ACE2 protein concentrations in the eye and brain ranged from approximately 2-16 ng/ml; ACE2 protein concentrations in the human heart and kidney have been previously reported to range between respectively, ~30 and ~100 ng/ml; https://www.abcam.com/human-ace2-elisa-kit-ab235649.html.

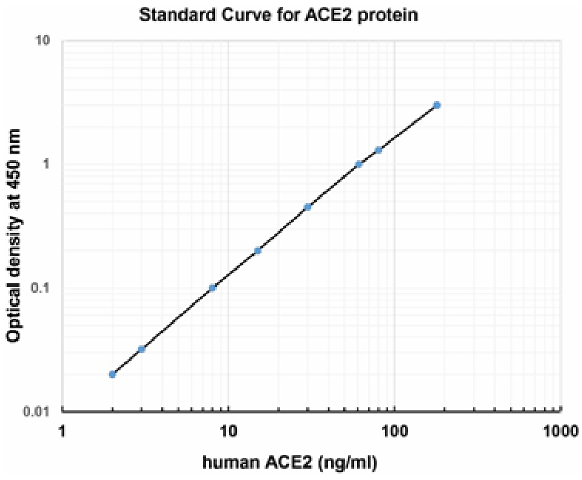

Supplementary File 1B

SUPPLEMENTARY FILE 1B - ELISA RESULTS - RAW DATA<br>(these MEAN +/- 1 S.D. data are graphed in Figure 2 in this paper)<br>ACE2 RECEPTOR PROTEIN IN THE VISUAL SYSTEM<br>(relative signal strength)	ELISA 1	ELISA 2	ELISA 3	MEAN	1 S.D.
whole brain	3.5	6	5.5	5	1.08
cerebral cortex	6	9.9	8.4	8.1	1.61
occipital lobe	14.1	11	10	11.7	1.7
temporal lobe	5.7	9	7.8	7.5	1.36
pons	13.5	15.9	18.6	16	2.08
cerebellum	4.1	3.8	5	4.3	0.51
optic nerve	9.1	10.8	11.9	10.6	1.15
whole eye	6.3	8.1	8.7	7.7	1.02
whole retina	9.9	7.4	9.4	8.9	1.08
corneal epithelial cells	1.7	1.5	1.3	1.5	0.16
retinal pigment epithelial cells	9.5	11.5	13.5	11.5	1.63
non-pigmented ciliary epithelial cells	5.4	6	6.9	6.1	0.62
trabecular meshwork cells	4.4	4	5.1	4.5	0.45
ocular choroid fibroblasts	6.4	8.9	7.8	7.7	1.02
